# Expression Microarray Analysis Reveals Alternative Splicing of *LAMA3* and *DST* Genes in Head and Neck Squamous Cell Carcinoma

**DOI:** 10.1371/journal.pone.0091263

**Published:** 2014-03-27

**Authors:** Ryan Li, Michael F. Ochs, Sun Mi Ahn, Patrick Hennessey, Marietta Tan, Ethan Soudry, Daria A. Gaykalova, Mamoru Uemura, Mariana Brait, Chunbo Shao, William Westra, Justin Bishop, Elana J. Fertig, Joseph A. Califano

**Affiliations:** 1 Department of Otolaryngology—Head and Neck Surgery, Johns Hopkins Medical Institutions, Baltimore, Maryland, United States of America; 2 Division of Oncology Biostatistics, Department of Oncology, Johns Hopkins Medical Institutions, Baltimore, Maryland, United States of America; 3 Milton J. Dance Head and Neck Center, Greater Baltimore Medical Center, Baltimore, Maryland, United States of America; 4 Department of Pathology, Johns Hopkins Medical Institutions, Baltimore, Maryland, United States of America; 5 Department of Mathematics and Statistics, The College of New Jersey, Ewing, New Jersey, United States of America; AC Camargo Cancer Hospital, Brazil

## Abstract

**Purpose:**

Prior studies have demonstrated tumor-specific alternative splicing events in various solid tumor types. The role of alternative splicing in the development and progression of head and neck squamous cell carcinoma (HNSCC) is unclear. Our study queried exon-level expression to implicate splice variants in HNSCC tumors.

**Experimental Design:**

We performed a comparative genome-wide analysis of 44 HNSCC tumors and 25 uvulopalatopharyngoplasty (UPPP) tissue samples at an exon expression level. In our comparison we ranked genes based upon a novel score—the Maximum-Minimum Exon Score (MMES) – designed to predict the likelihood of an alternative splicing event occurring. We validated predicted alternative splicing events using quantitative RT-PCR on an independent cohort.

**Results:**

After MMES scoring of 17,422 genes, the top 900 genes with the highest scores underwent additional manual inspection of expression patterns in a graphical analysis. The genes *LAMA3, DST, VEGFC, SDHA, RASIP1*, and *TP63* were selected for further validation studies because of a high frequency of alternative splicing suggested in our graphical analysis, and literature review showing their biological relevance and known splicing patterns. We confirmed *TP63* as having dominant expression of the short *DeltaNp63* isoform in HNSCC tumor samples, consistent with prior reports. Two of the six genes (*LAMA3* and *DST*) validated by quantitative RT-PCR for tumor-specific alternative splicing events (Student's t test, P<0.001).

**Conclusion:**

Alternative splicing events of oncologically relevant proteins occur in HNSCC. The number of genes expressing tumor-specific splice variants needs further elucidation, as does the functional significance of selective isoform expression.

## Introduction

Head and neck squamous cell carcinoma (HNSCC) represents the sixth most prevalent solid tumor reported annually [1]. These tumors predominantly arise from the epithelia of the upper aerodigestive tract. Prognostically HNSCC is a heterogeneous group with known clinicopathologic predictors of treatment response including TNM staging, tobacco consumption status, and presence of high-risk HPV infection [2]–[7]. The biological underpinnings explaining HNSCC tumorigenesis and diverse tumor behavior remain an area of ongoing investigation.

Recent advances in understanding the genetic mutational landscape of HNSCC have shed light upon upregulated oncogenic and disrupted tumor suppressor pathways contributing to this disease. The most prevalent mutations in HNSCC include *TP53* and *NOTCH1* genes, found in ∼50% and 15% of HNSCC tumors respectively [8], [9]. In addition to somatic mutations, additional drivers of tumorigenesis have been elucidated in HPV-associated oropharyngeal HNSCC, wherein human papilloma (HPV) viral oncogenic expression promotes degradation of important cell-cycle regulators such as *retinoblastoma (Rb)*, thereby abrogating a critical tumor suppressor pathway [10].

Another avenue of gene expression regulation in cancer that is relatively under-investigated is alternative splicing—wherein the post-trascriptional modification of mature mRNA greatly increases the functional diversity of gene products. Widespread changes in splicing patterns have been demonstrated when comparing tumor and normal epithelial tissues of the breast and ovaries [11]. How these prevalent alternative splicing events are implicated in tumorigenesis is not presently clear.

Similar genome-wide studies of alternative splicing in HNSCC have not yet been reported. There is reason to believe that selective isoform expression may be relevant in HNSCC development. For example *TP63* is a member of the *p53* superfamily from which six major isoforms are transcribed—three short and three long isoforms share common upstream promoters. The short *DeltaNp63* isoform is dominant in both normal epithelial cells and HNSCC, and furthermore *DeltaNp63* appears to be selectively overexpressed in HNSCC tumor cells [12], [13]. Another *p53* superfamily protein, *p73*, believed to mediate apoptosis in response to DNA damage, may be inactivated by interaction with the *DeltaNp63* isoform as was shown previously [14]. This mechanism may demonstrate how differential isoform expression can promote tumorigenesis in the absence of a genetic mutation.

Various technologies have been employed in the detection of alternative splicing events. High-throughput quantitative RT-PCR utilizes massive libraries of isoform-specific primers [11]. Expression microarray platforms interrogate whole-transriptome mRNA via sequence-specific probes, and can provide exon-level expression data for comparison of known or putative isoform expression between samples [15]–[18]. RNA-seq uses deep sequencing techniques to characterize the entire transcriptome complete with quantitative description of specific isoform expression [19]. Regardless of the technology, it can be difficult to define and identify alternative splicing events. For example in an individual gene, expression of two isoforms in equal proportions may carry biological significance, whereas for another gene, expression of one isoform at a tenth of the level of a dominant isoform may carry great physiological importance. The maximum-minimum exon scoring model (MMES) developed in our study rewards large differences in exon expression levels within a single gene with higher scores, as a strategy for predicting alternative splicing.

Various approaches to exon array analysis have been utilized in the detection of alternative splicing events. Many studies utilize a variant of the splicing index calculation [17], [20]. This approach compares the signal of an individual probeset—relative to a summary signal of the corresponding gene—between two or more groups. The gene level summary signal is typically derived from averaging of probeset signals across the gene. The accuracy of this summary signal is crucial, as its value relative to an individual probeset signal is a key determinant of the splicing signal's predictive value. The variation of reliability weighted fold change (VFC) is another recent approach that analyzes the range of probeset values across the gene, and regards large intragenic probeset signal spreads as suggestive of alternative splicing. Our MMES model has elements of both the splicing index and VFC algorithms. MMES is sensitive to large ranges in maximum and minimum exon expression signals, as in the VFC model. Like the splicing index model, MMES compares individual probeset signals within a tumor to the signal within a normal, or different cohort. MMES differs, in that the comparison to normal probeset signals is for normalization of the tumor probeset signal, prior to calculating the maximum-minimum range of exon signals. This range is the metric that is ultimately used for ranking genes, as opposed to an index or ratio of tumor-to-normal probeset signals.

In this current investigation we employed the Affymetrix Human Exon 1.0ST mRNA expression array to detect alternative splicing events and differential isoform expression specific to HNSCC tumors in comparison to normal upper aerodigestive tract control tissues. We describe our methods for expression microarray analysis at an exon-level and results of validation studies to confirm tumor-specific splice variant expression of genes contributing to the cell-adhesion and cytoskeletal properties of cells.

## Materials And Methods

**Figure 1:**
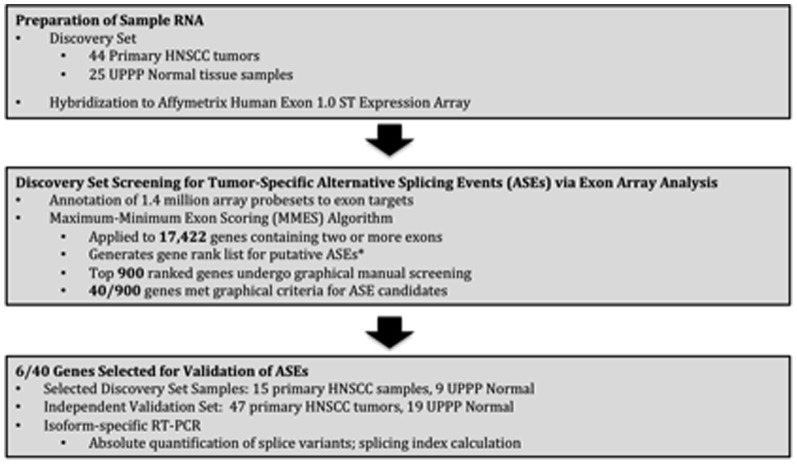
Figure 1 demonstrates the experimental flow from tissue procurement to validation of tumor-specific alternative splicing events in our study.

Figure 1 demonstrates the experimental flow from tissue procurement to validation of tumor-specific alternative splicing events in our study.

### Human Tissue Samples

Written consent was obtained from all participants for the procurement and study of human HNSCC tissue samples and normal mucosal tissues. The Johns Hopkins Medical Institutions (JHMI) institutional review board (IRB) approved this study and the consent procedure. Copies of all written consents are maintained by JHMI. Tissues were snap frozen in liquid nitrogen immediately after collection. Microdissection of frozen tissue was performed to assure that more than 75% of tissue contained HNSCC. Microdissection was performed under supervision of Johns Hopkins Hospital head and neck pathologists (W.W and J.B.). For the discovery set, 44 samples of HNSCC and 25 samples of normal oropharyngeal epithelial tissue were used for the Affymetrix Human Exon 1.0 ST mRNA expression array. A separate validation set of 47 microdissected HNSCC tumors and 19 normal oropharyngeal epithelial tissues was used for the validation studies. All normal tissues were derived from patients who underwent uvulopalatopharyngoplasty for obstructive sleep apnea.

### RNA Extraction

Total RNA extraction from human tissue samples was performed using Trizol reagent (Invitrogen, Carlsbad, Calif., USA) using standard methods [21], [22].

### RNA Analysis on the Affymetrix Human Exon 1.0ST Array

RNA from discovery set samples was labeled using the Whole Transcript Sense Target Labeling protocol described by Affymetrix and reagent from Ambion and Affymetrix. Briefly, 100 ng of total RNA was used to synthesize first strand cDNA using random oligonucleotides with T7 promoter as primer and the SuperScript Choice System (Invitrogen, Carlsbad, California). Following the double stranded cDNA synthesis, the double strand cDNA was purified using magnetic beads, and cRNA was generated through in vitro transcription. 10 ug of cRNA was then used to generate sense strand cDNA using random primer, dNTP-dUTP mix, and Superscript II reverse transcriptase (Invitrogen, Carlsbad, California). The resulting sense strand cDNA was then purified, fragmented using UDG and APE at 37 C for 60 minutes, and terminal labeled with biotinylated nucleotide and terminal DNA transferase at 37 C for 60 minutes. The labeled sense strand DNA was hybridized to the Affymetrix GeneChip human Exon 1.0 ST arrays for 17 hr at 45 C with constant rotation (60 rpm). Affymetrix Fluidics Station 450 was used to wash and stain the Chips, removing the non-hybridized target and incubating with a streptavidin-phycoerythrin conjugate to stain the biotinilated cDNA. The staining was further amplified using goat IgG as blocking reagent and biotinilated anti-streptavidin antibody (goat), followed by a second staining step with a streptavidin-phycoerythrin conjugate. Fluorescence was detected using the Affymetrix G3000 GeneArray Scanner and image analysis of each GeneChip was perfomed through the Affymetrix GeneChip Command Console version 3.4 (AGCC v3.4) software from Affymetrix. Data from this discovery set is available on the Gene Expression Omnibus, GSE33205.

This array interrogates the expression of gene transcripts on a whole-genome level. Over 1.4 million array probesets are designed to target exonic sequences within the human genome based upon a prediction model of exon boundaries described in the Affymetrix GeneChip Technical Note [15]. In general, probesets are designed to interrogate exonic sequences, without overlap in targeted sequences between probesets. We selected core probesets for analysis. Core probesets target genomic exonic sequences with the highest level of confidence based upon their design using RefSeq data and full-length mRNA annotations.

### Array Analysis for Alternative Splicing Events

**Figure 2:**
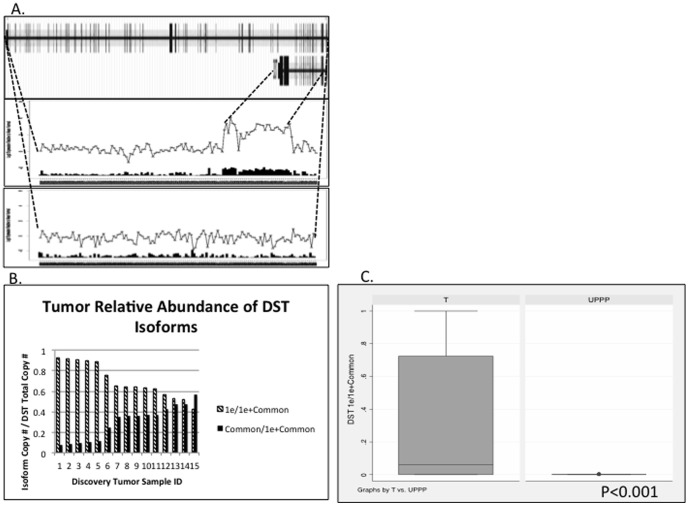
Splice variants of *DST* demonstrated in exon array analysis (A and B) and quantitative RT-PCR validation (C and D). A, Example of discovery HNSCC primary tumor samples (25016 and 25579) demonstrating dominant expression of *1e* and *1eA* isoforms, respectively. Uppermost box displays all exons for both isoforms of *DST*. Middle and lower boxes display exon-specific array probesets along horizontal axis, and probeset signal relative to normal (0) on the vertical axis, *log_2_ scale*. For each probeset, the inset histograms display the number of standard deviations from the mean probeset signal in UPPP samples, that the probeset signal deviates in each individual tumor. B, Splice index calculations [Absolute Copy # of *DST* isoform / (*1e + 1eA*)] in 15 discovery tumor samples. C, Comparison of mean splicing index for *DST* isoforms in an independent validation set of 47 HNSCC tumors vs. 19 UPPP samples, *two-tailed Student’s t test, p<0.0001*.

Affymetrix array core probeset data was normalized with the Robust Multiarray Average (RMA) method using the Bioconductor oligo package in R [23]. We apply this new approach, the Maximum-Minimum Exon Scoring (MMES) model, to rank genes for the presence of potential alternative splicing events. While some genes with only one annotated probeset (and presumably one exon) may truly exhibit alternative splicing events at novel splice sites, we limited our analysis to genes with at least three probesets (17,422 genes), for comparison of intragenic probeset signals in the MMES model. The flow of the model is as follows:

1. 1] After RMA normalization of all core probesets, every probeset signal in each individual tumor is normalized to the mean respective probeset signal in the normal samples, generating a value P_itN_ (i =  probeset ID, t =  tumor ID, N =  normalized) for each tumor.
2. 2] Many exons are interrogated by more than one probeset. A mean of all P_itN_ values for an individual exon is calculated for each tumor, generating a value Exon_et_ (e =  exon ID, t =  tumor ID).
3. 3] In each tumor, for each gene the difference between the maximum Exon_et_ and minimum Exon_et_ is calculated, generating one MMES score per gene, per tumor. A mean tumor MMES score for each gene can be calculated, and a rank list generated with higher mean tumor scores suggesting putative alternative splicing events. Larger differences in the relative expression levels of exons within a single gene are rewarded with higher MMES scores. Greater frequency of high MMES scores within the tumor cohort for a particular gene are reflected by a higher mean MMES score, and consequently ranked more highly.
4. 4] From the MMES generated rank list (Table S2), the top 900 scoring genes were further subjected to a visual screening process. Visual screening begins with a graphical comparison of the discovery tumor cohort and UPPP sample expression array data. *DST* gene expression data is used as an example (Figure S4C). All core probesets annotated to *DST* are presented on the horizontal axis from 5′ (left) to 3′ (right) end. The vertical axis represents the RMA normalized probeset expression signal on a log_2_ scale. The flat horizontal tracing represents the mean signal in all probesets across both tumors and normals rounded down to the nearest whole number and merely provides a convenient horizontal reference. The irregular tracing represents for each probeset the difference in the means of the tumor and normal samples relative to the horizontal line indicating zero. This provides a view of the difference shifted to the bulk of the individual data points. The histogram in Figure S4C describes the number of tumor samples that have probeset signals one standard deviation greater (or less) than the corresponding mean probeset signal in the UPPP cohort. The graphical representation in Figure S4C provides an overview of differential exonal expression between the tumor and UPPP cohorts that may suggest alternative splicing. All probesets can be mapped to corresponding exons (Figure S4C top) using publicly available annotation maps from the human genome 18 (hg18) assembly. Because meaningful alternative splicing may occur in a subset of tumor samples, manual graphical screening of individual tumors is then necessary. Figure 2A illustrates this process. Two individual tumor plots are illustrated. Again with the exon annotation displayed above. The horizontal axis of the tumor plots again displays all probesets for *DST*. The vertical axis here represents a select tumor's relative probeset expression compared to the mean for that probeset of the UPPP cohort that is represented by the zero level. By examining individual tumor plots we identify putative alternative splicing events that occur in a significant number of individual samples with less ‘noise’ than may be seen from overall comparisons of tumor and UPPP cohorts.

### Validation of Alternative Splicing by Quantitative RT-PCR

We selected 15 tumors from the discovery set for validation of select alternative splicing candidates from our MMES prediction model. For each selected tumor, 2 micrograms of RNA was converted to cDNA using the Quanta Biosciences qScript cDNA SuperMix using standard methods, producing a total of 20 microliters of cDNA [24]. A 5-fold dilution of cDNA with DNAse and RNAse-treated sterile water was performed to conserve finite tumor RNA, for a final cDNA concentration of 20 ng/microliter in 100 microliters total volume. Conversion of RNA to cDNA from the 47 tumors and 19 UPPP tissues samples of the validation set was performed in an identical fashion.

Quantitative RT-PCR was performed using custom primer and probe sets specific to each splice variant (Table S1.), as well as 18 S as a housekeeping gene. 30 nanograms of cDNA was run in an Applied Biosystem Taqman 7900 HT real-time PCR machine. Splice-variant specific amplicons were generated using splice variant-specific primers in conventional PCR with JHU 011 and Fadu cell line RNA templates. Serial 10-fold dilutions of amplicons were used for standards during quantitative RT-PCR for absolute quantification of splice variant copy number.

To compare splicing patterns between tumor samples and UPPP samples, splicing indices were calculated for the each gene as:
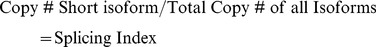



Data was analyzed using Stata v11.0 statistical analyses software (Stata Corporation, College Station, TX, USA). Splicing Indices were compared between tumor and UPPP normal tissue cohorts for significant differences using 2-tailed Student's t test with assumption of unequal variance.

## Results

The clinicopathologic characteristics of discovery and validation tumors and UPPP normal samples are presented in Tables 1, 2, 3, and 4. All tumors were squamous cell carcinoma of the upper aerodigestive tract originating in subsites of the oral cavity, oropharynx, or larynx. Tobacco and alcohol exposure history, and tumor staging are also reported.

**Table 1:** Demographic characteristics of discovery tumor and UPPP normal tissue samples.

Demographics			
	Tumor n = 44	Normal n = 25	P-value
Mean Age (Years)	57.2	29	P<0.0001
Gender			
Male	31	10	P = 0.013
Female	13	15	
Race			
White	40	14	P = 0.001
Black	3	11	
Other	1	0	
Tobacco			
Yes	29	3	P<0.001
No	14	22	
Unknown	1	0	
Alcohol			
Yes	19	3	P<0.001
No	24	22	
Unknown	1	0	

**Table 2:** Discovery tumor clinical characteristics.

Tumor Staging	
**Tumor Site**	
Oral Cavity	11
Oropharynx	20
Larynx	13
**T Stage**	
1	14
2	11
3	7
4	11
Unknown	1
**N Stage**	
0	15
1	5
2a	6
2b	14
2c	4
3	0
**M Stage**	
0	44
1	0
**TNM Stage**	
I/II	2
III/IV	42

**Table 3:** Demographic characteristics of validation tumor and UPPP normal tissue samples.

Demographics			
	Tumor n = 47	Normal n = 19	P-value
Mean Age (Years)	59.3	31.6	P<0.0001
Gender			
Male	41	7	P<0.001
Female	6	12	
Race			
White	33	13	P = 0.824
Black	9	3	
Other	5	3	
Tobacco			
Yes	31	7	P = 0.012
No	13	12	
Unknown	3	0	
Alcohol			
Yes	18	1	P = 0.005
No	26	18	
Unknown	3	0	

**Table 4:** Validation tumor clinical characteristics.

Tumor Staging	
**Tumor Site**	
Oral Cavity	11
Oropharynx	20
Larynx	16
**T Stage**	
1	6
2	18
3	14
4	9
**N Stage**	
1	17
2a	10
2b	1
2c	16
3	3
**M Stage**	
0	47
1	0
**TNM Stage**	
I/II	0
III/IV	47

Using our prediction model for alternative splicing events, 17,422 genes were ranked by MMES score (Table S1). We subsequently performed a manual gene plot review of the top 900 ranked genes to identify the strongest candidates amongst this group for differential alternative splicing between tumors and UPPP normal samples. These plots are available for download in PDF format from the online supplement. From these 900 candidate genes, 40 were designated as strong candidates for differential alternative splicing after manual gene plot review. We reviewed the literature on the biological relevance of these 40 genes, and selected six genes from the manual gene plot review that demonstrated high likelihood of validating alternative splicing events. These six genes included *LAMA3A, DST, RASIP1, SDHA, VEGFC*, and *TP63*. Table 5 summarizes the chromosomal location, function, and pathologic implications reported for these genes in the literature. For each gene, we attempted quantitative RT-PCR validation of 15 selected discovery tumors followed by verification of alternative splicing events in 47 tumors and 19 UPPP normal samples from the validation set, where we were able to validate both *LAMA3* and *DST*. For the *TP63* gene we confirmed a markedly predominant expression of the shorter *DeltaNp63* isoform that has been previously reported for epithelia of the upper aerodigestive tract [12], [13]. We include these results here to demonstrate the detection of splice variants by way of expression array analysis. *RASIP1, SDHA, VEGFC* confirmatory RT-PCR studies did not validate alternative splicing events predicted from microarray analysis and results are summarized in the Supporting Information (Figures S1, S2, and S3). For all samples tested, all results have been normalized to *18 S* expression.

**Table 5:** Descriptive summary of genes selected for quantitative RT-PCR validation of alternative splicing events.

Gene Name	Chromosome	# Ref Seq Isoforms	Protein Function	Pathologic Implications
*LAMA3*	chr18:21,452,984-21,535,029	4	Subunit of Laminin-332: extracellular linker between BM and cell-surface integrins	Overexpression of laminin-332 in squamous cell carcinomas promotes invasion
*DST*	chr6:56,322,785-56,507,694	2	Links intracellular cytoskeleton to extracellular hemidesmosomes	Implicated in bullous pemphigoid
*RASIP1*	chr19:49,223,842-49,243,970	1	Essential role in vascular endothelial development	Unknown
*SDHA*	chr5:218,356-256,814	1	Krebs cycle cell metabolism protein subunit	Somatic mutations described in paraganglioma and GIST*
*VEGFC*	chr4:177,604,691-177,713,895	1	Ligand to the VEGFR3 receptor	Implicated in angiogenesis in breast cancer
*TP63*	chr3:189,349,216-189,615,068	6	Inhibits p73-mediated apoptosis, promoting cell survival	DeltaN isoform is overexpressed in HNSCC

### *TP63* Isoform Expression

**Figure 3:**
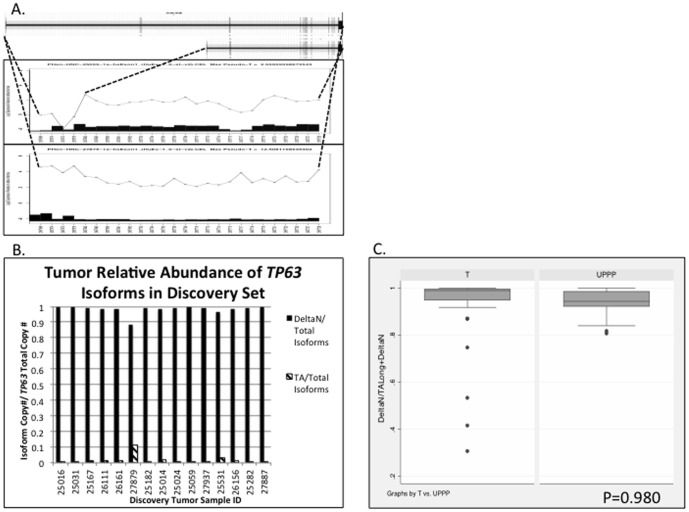
Splice variants of *TP63* demonstrated in exon array analysis (A) and quantitative RT-PCR validation (B and C). A, Example of discovery HNSCC primary tumor samples predicted to have dominant expression of *TAp63* (long) and *DeltaNp63* (short) isoforms, respectively. Uppermost box displays all exons for long and short isoforms of *TP63*. Middle and lower boxes display exon-specific array probesets along horizontal axis, and probeset signal relative to normal (0) on the vertical axis, *log_2_ scale*. For each probeset, the inset histograms display the number of standard deviations from the mean probeset signal in UPPP samples, that the probeset signal deviates in each individual tumor. B, Splice index calculations [Absolute Copy # of *TP63* isoform / (*TAp63+ DeltaNp63*)] in 15 discovery tumor samples. C, Comparison of mean splicing index for *TP63* isoforms in an independent validation set of 47 HNSCC tumors vs. 19 UPPP samples, *two-tailed Student’s t test, P = 0.980*.

*TP63* RefSeq annotations demonstrate six major isoforms of this gene. Three longer isoforms (*TAp63*) share ten consecutive exons from the 5′-end (*TP63 TA isoforms* NM_001114979.1, NM__001114978.1, and NM_003722.4). Three shorter isoforms (*DeltaNp63*) share eight consecutive exons from the 5′-end (*DeltaNp63 isoforms*, NM__001114980.1, NM__001114982.1, NM__001114981.1). In our exon array analysis, manual review of *TP63* gene plots predicted 25 of 44 tumor samples with a splicing pattern predominantly expressing the shorter *DeltaNp63* isoforms. Figure 3 demonstrates select individual tumors with either *DeltaNp63* or *TAp63* predominant expression (Figure 3A), and a *TP63* gene plot comparing all tumors to UPPP normal samples (Figure S4A), predicting that overall tumors have a higher predominance of *DeltaNp63* isoform expression.

Subsequent quantitative RT-PCR validation of 15 discovery set tumors showed dominant expression of the *DeltaNp63* isoforms (Figure 3B). Our independent validation set further demonstrated dominant expression of the *DeltaNp63* isoform in both tumors and UPPP samples so that there was no difference in the relative proportion of *DeltaNp63* and *TAp63* isoform expression between tumor and UPPP samples (*two-tailed Student's t test, P = 0.980*, Figure 3C). Quantitative RT-PCR however did demonstrate overexpression of the *DeltaNp63* isoform in tumors relative to UPPP samples within the validation set (*two-tailed Student's t test, P = 0.006*), consistent with prior reports in literature [12], [13], [25].

### Alternative Splicing of *LAMA3*

**Figure 4:**
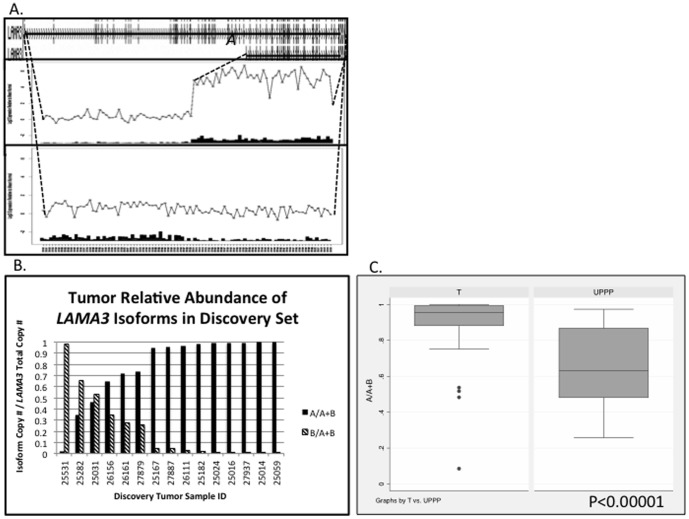
Splice variants of *LAMA3* demonstrated in exon array analysis (A) and quantitative RT-PCR validation (B and C). A, Example of discovery HNSCC primary tumor samples (25059 and 25282) demonstrating dominant expression of *LAMA3A* and *LAMA3B* isoforms, respectively. Uppermost box displays all exons for both isoforms of *LAMA3*. Middle and lower boxes display exon-specific array probesets along horizontal axis, and probeset signal relative to normal (0) on the vertical axis, *log_2_ scale*. For each probeset, the inset histograms display the number of standard deviations from the mean probeset signal in UPPP samples, that the probeset signal deviates in each individual tumor. B, Splice index calculations [Absolute Copy # of *LAMA3A* isoform / (*LAMA3A + LAMA3B*)] in 15 discovery tumor samples. C, Comparison of mean splicing index for *LAMA3* isoforms in an independent validation set of 47 HNSCC tumors vs. 19 UPPP samples, *two-tailed Student’s t test, p<0.0001*.

*LAMA3* RefSeq annotations demonstrate four major isoforms of this gene, with the two shorter isoforms (*LAMA3A* NM_001127718.1 and NM_000227.3) excluding the 36 consecutive most 5′ exons, and full-length *LAMA3B* variants (*LAMA3B* NM_001127717.1 and NM_198129.1). In our exon array analysis, manual review of *LAMA3* gene plots demonstrated 23 of 44 tumor samples with a splicing pattern predominantly expressing the shorter *LAMA3A* isoforms. Figure 4 demonstrates select individual tumors with either *LAMA3A* or *LAMA3B* predominant expression (Figure 4A), and a *LAMA3* gene plot comparing all tumors to UPPP normal samples (Figure S4B), predicting that overall tumors have a higher predominance of *LAMA3A* short isoform expression.

Subsequent quantitative RT-PCR validation of 15 discovery set tumors corroborated the gene plot predictions of either *LAMA3A* or *LAMA3B* predominant expression (Figure 4B). Our independent validation set further confirmed that in tumors, *LAMA3A* isoforms comprised a significantly greater proportion of all *LAMA3* transcripts as compared to UPPP normal samples (*two-tailed Student's t test, p<0.0001*, Figure 4C).

### Alternative Splicing of *DST*

*DST* RefSeq annotations demonstrate five major isoforms for this gene. Our manual review of *DST* gene plots predicted predominant expression of either the short isoform (*1e*, NM__001723.5)—excluding the eight most 3′ exons, and including a long sequence that is intronic and not transcribed in the longer isoforms—or one of four longer isoforms that we probed with a ‘common’ primer/probeset (*1eA*, NM__183380.3; *Isoform 3* NM_001144770.1; *Isoform 2*, NM__001144769.2; *Isoform 1*, NM__183380.3). In our exon array analysis, manual review of *DST* gene plots demonstrated 14 of 44 tumor samples with a splicing pattern predominantly expressing the shorter *1e* isoform. Figure 3 demonstrates select tumors with either *1e* or longer isoform predominant expression (Figure 2A), and a *DST* gene plot comparing all tumors to UPPP normal samples (Figure S4C), showing that overall tumors have a higher predominance of *1e* short isoform expression.

Subsequent quantitative RT-PCR validation of 15 discovery set tumors corroborated the gene plot predictions of either *1e* or longer isoform predominant expression (Figure 2B). Our independent validation set demonstrated that in tumors, *1e* comprised a significantly greater proportion of all *DST* transcripts as compared to UPPP normal samples (*two-tailed Student's t test, p<0.0001*, Figure 2C).

## Discussion

The expression of multiple unique transcripts from a single genetic sequence can greatly increase the influence of a single gene on diverse biological processes [26]. Alternative splicing may be relevant in the pathophysiology of various cancers as well. For the gene *TP63* our results were consistent with prior work reporting a predominance of *DeltaNp63* isoform expression in malignant-transformed upper aerodigestive tract epithelium. In this study we utilized an exon expression microarray platform to detect tumor-specific alternative splicing events in head and neck squamous cell carcinoma, and found a significant difference in the transcriptional pattern of *LAMA3* and *DST*, two genes involved in cell adhesion and cytoskeletal structure respectively. This is consistent with prior research in squamous cell carcinoma suggesting a shift in isoform expression patterns for these two genes.

The potential oncologic role for alternative splicing in HNSCC has not been well characterized. Alternative splicing may significantly influence the transcriptional milieu in HNSCC as it does in other tumor types. Large genome-wide studies of ovarian and breast cancer illuminate the common occurrence of alternative splicing in the post-transcriptional processing of mRNA. Venables et al 2009 designed a massive RT-PCR screening primer library for all alternative splicing events annotated in the RefSeq database, totaling 2,168 [11], [27], [28]. The metric used in this study to describe prevalence of individual isoforms was analogous to that in our study—[concentration of long isoform]/[concentration of all isoforms expressed]. The authors defined alternative splicing as occurring if the proportion of two or more isoforms each was greater than 10% of the denominator. Of greater than 600 alternative splicing events detected in both ovarian and breast samples, over half of these patterns were reversed in ovarian and breast cancer samples relative to respective normal tissues, emphasizing a broad change in splicing patterns that evolved in tumor development. In addition to using changes in splicing patterns as biomarkers for cancer, the need for functional investigations is clear.

In various other solid tumors including lung and colon cancer, the Affymetrix Human Exon 1.0ST expression microarray platform has been used to identify alternative splicing events that differ between normal and cancer tissue [17], [18], albeit on a much more limited set of genes than detected on Venables et al's RT-PCR screening platform. Often at the validation stage of putative cancer-specific alternative splicing, five to ten genes remain for which RT-PCR validation corroborates array predictions. The difficulty of detecting splice variants via array analysis is apparent in these studies. The flow of basic array analysis for detecting alternative splicing events in cancer requires: 1] quality control analysis of raw array data; 2] normalization of probeset level data across multiple sample arrays; 3] a semi-quantitative comparison of exon expression between tissue types (cancer vs. normal); 4] manual review of exon-level data often in graphical format; 5] followed by validation of array predictions [18]. Step 3 generates a gene rank list of putative alternative splicing events, whereas Step 4 manually selects from this rank list candidates most likely to validate, with knowledge of the annotated structure of the gene and known splice variants. In expression microarray comparison of cancerous and normal tissues, no strict threshold exists after which one can predict with absolutely certainty that the splicing pattern has changed until the validation step. Most often expression of one isoform does not imply complete suppression of the other; rather both comprise a proportion of a particular gene's transcripts.

Few recent studies have discussed the importance of this process in HNSCC, and the functional significance is even less understood [29], [30]. Despite the promise of genome-wide analysis via expression microarray to detect alternative splicing events, few studies have validated events in more than two or three genes in HNSCC. We likewise have validated the differential expression of *LAMA3* and *DST* isoforms in HNSCC compared to normal tissues.

In comparing tumor and normal upper aerodigestive tract tissues, our expression microarray analysis suggested a quantitative difference in expression levels of select isoforms for several genes known to be involved in cell adhesion processes, of particular interest in cancer studies. Microarray design relies upon accurate oligonucleotide probe designs, targeting unique exon sequences or exon-exon junctions—for the array used in this study, junctional probes were not available. Probe-based gene expression profiling has intrinsic limitations, including the requirement to have prior knowledge of exonic sequences for design, the risk of cross-hybridization to non-specific targets, a potentially non-linear dynamic range of probe signals relative to expression levels, and background signals that may be difficult to overcome. Newer RNA-seq technologies can sequence entire cell transcriptomes with high accuracy, and potentially quantify individual isoform expression levels [19]. The deep-sequencing technology used here does not rely upon sequence-specific probes. Consequently RNA-seq may more precisely characterize novel isoforms for which exon-exon boundaries are not known with certainty. Significant challenges remain in RNA-seq analysis—accurate alignment of sequence reads is an evolving area of research, as is the accurate quantification of individual exon expression levels, as coverage may not be uniform even over an individual gene [31].

*LAMA3* isoforms comprise the alpha subunit of several laminin-class heterotrimeric, extracellular proteins within the basement membrane of epithelial linings of the upper aerodigestive tract, intestinal lining, skin, lungs, and cervix [32]. The *LAMA3A* isoform is a subunit of *laminin 332*, which is highly expressed in squamous cell carcinomas, and may be associated with reduced patient survival as well as increased tumor invasiveness [33], [34]. The *LAMA3B* isoform comprises the alpha subunit of another *laminin (3B32)* that has not been implicated in squamous cell carcinoma to date. The *LAMA3A* isoform lacks the N-terminal domain (LN) of the full-length *LAMA3* transcript *LAMA3B*. The LN domain is necessary for the polymerization of laminins and promotion of epithelial basement membrane stability [35]. Both isoforms contain the globular C-terminal domains necessary for interactions with extracellular membrane integrins that can affect intracellular signaling cascades. How preferential expression of *LAMA3A* in HNSCC affects tumor invasiveness requires further investigation.

A recent study compared *LAMA3* expression in HNSCC tumors that were scored as having high versus low hypoxic microenvironments based upon a 99-gene hypoxia expression panel [36]. In tumors with higher hypoxia scores, expression of the *LAMA3A* isoform was greater compared to tumors with low hypoxia scores. However the *LAMA3B* isoform was not differentially expressed. HNSCC cell lines subjected to hypoxic conditions did not replicate the hypoxia-associated increase in *LAMA3A* expression. This study focused on differential expression of *LAMA3* isoforms in biologically different tumors. Our results compare primary HNSCC tumors to normal tissue and confirm a tumor-specific predeliction for *LAMA3A* isoform expression. The pathophysiological implications of *LAMA3A* overexpression, and characterization of laminin 332 expression in HNSCC is a new area of interest.

*DST* encodes the sequence for the cytoskeletal bullous pemphigoid antigen 1 (*BPAG1*) protein that has multiple isoforms variably expressed in different tissue types [37],[38]. Keratinocytes predominantly express the cytoplasmic-expressed *1e* isoform, with lower expression levels of alternative isoforms. While *BPAG1* mutations are implicated in bullous pemphigoid blistering diseases, little is known about a potential role in cancer [39]. Like *LAMA3A*, isoform *1e* interacts with the critical integrin *alpha6beta4* that facilitates keratinocyte adhesion to the basement membrane [40]. mRNA and protein expression studies using in-situ hybridization and indirect immunofluorescence respectively have shown both 5 to 10-fold increased expression of the *1e* isoform in head and neck squamous carcinoma compared to normal upper aerodigestive tract epithelial cells, and aberrant delocalization of this protein away from the basal membrane, the usual site of hemidesmosomal expression [40]. In squamous carcinomas both *alpha6beta4* integrin and *1e* protein expression was distributed along all cell membranes, having lost their polarized expression. In addition, diminished hemidesmosomal formation was seen on electron microscopic analysis of the same tumors, with apparent greater loss in metastatic tumors [39]. Our study corroborates increased *1e* mRNA expression in a large series of HNSCC tumors compared to normal controls.

In conclusion alternative splicing events of oncologically relevant proteins do occur in HNSCC. The number of genes expressing tumor-specific splice variants needs further elucidation, as does the functional significance of selective isoform expression.

## Supporting Information

Figure S1 ***VEGFC*** **.**
(TIFF)

Figure S2 ***SDHA*** **.**
(TIFF)

Figure S3 ***RASIP1*** **.**
(TIFF)

Figure S4 **Visual screening begins with a graphical comparison of the discovery tumor cohort and UPPP sample expression array data.** A,*TP63 gene*. Comparing the mean expresssion of each probeset in the entire discovery tumor set to the mean in the entire discovery UPPP set. The mean probeset signals for the UPPP sample set have been zeroed (horizontal line), with adjustment of tumor cohort mean probeset signals (oscillating line), *log_2_ scale*. B, *LAMA3 gene*. Comparing the mean expresssion of each probeset in the entire discovery tumor set to the mean in the entire discovery UPPP set. The mean probeset signals for the UPPP sample set have been zeroed (horizontal line), with adjustment of tumor cohort mean probeset signals (oscillating line), *log_2_ scale*. C, *DST gene*. Comparing the mean expresssion of each probeset in the entire discovery tumor set to the mean in the entire discovery UPPP set. The mean probeset signals for the UPPP sample set have been zeroed (horizontal line), with adjustment of tumor cohort mean probeset signals (oscillating line), *log_2_ scale*.
(TIFF)

Table S1 **Splice variant-specific custom primer and probe sequences.**
(XLSX)

Table S2 **MMES Gene Rank List.**
(XLSX)

## References

[1] Leemans CR, Braakhuis BJ, Brakenhoff RH (2011) The molecular biology of head and neck cancer. Nat Rev Cancer 11: 9–22. 21160525 10.1038/nrc2982

[2] Ang KK, Harris J, Wheeler R, Weber R, Rosenthal DI, et al (2010) Human papillomavirus and survival of patients with oropharyngeal cancer. N Engl J Med 363: 24–35. 20530316 10.1056/NEJMoa0912217 PMC2943767

[3] Fakhry C, Westra WH, Li S, Cmelak A, Ridge JA, et al (2008) Improved survival of patients with human papillomavirus-positive head and neck squamous cell carcinoma in a prospective clinical trial. J Natl Cancer Inst 100: 261–269. 18270337 10.1093/jnci/djn011

[4] Gillison ML, Zhang Q, Jordan R, Xiao W, Westra WH, et al (2012) Tobacco smoking and increased risk of death and progression for patients with p16-positive and p16-negative oropharyngeal cancer. J Clin Oncol 30: 2102–2111. 22565003 10.1200/JCO.2011.38.4099 PMC3397696

[5] Lassen P, Eriksen JG, Krogdahl A, Therkildsen MH, Ulhoi BP, et al (2011) The influence of HPV-associated p16-expression on accelerated fractionated radiotherapy in head and neck cancer: evaluation of the randomised DAHANCA 6&7 trial. Radiother Oncol 100: 49–55. 21429609 10.1016/j.radonc.2011.02.010

[6] Rischin D, Young RJ, Fisher R, Fox SB, Le QT, et al (2010) Prognostic significance of p16INK4A and human papillomavirus in patients with oropharyngeal cancer treated on TROG 02.02 phase III trial. J Clin Oncol 28: 4142–4148. 20697079 10.1200/JCO.2010.29.2904 PMC2953971

[7] Fortin A, Wang CS, Vigneault E (2009) Influence of smoking and alcohol drinking behaviors on treatment outcomes of patients with squamous cell carcinomas of the head and neck. Int J Radiat Oncol Biol Phys 74: 1062–1069. 19036528 10.1016/j.ijrobp.2008.09.021

[8] Agrawal N, Frederick MJ, Pickering CR, Bettegowda C, Chang K, et al (2011) Exome sequencing of head and neck squamous cell carcinoma reveals inactivating mutations in NOTCH1. Science 333: 1154–1157. 21798897 10.1126/science.1206923 PMC3162986

[9] Stransky N, Egloff AM, Tward AD, Kostic AD, Cibulskis K, et al (2011) The mutational landscape of head and neck squamous cell carcinoma. Science 333: 1157–1160. 21798893 10.1126/science.1208130 PMC3415217

[10] Gillison ML, Koch WM, Capone RB, Spafford M, Westra WH, et al (2000) Evidence for a causal association between human papillomavirus and a subset of head and neck cancers. J Natl Cancer Inst 92: 709–720. 10793107 10.1093/jnci/92.9.709

[11] Venables JP, Klinck R, Koh C, Gervais-Bird J, Bramard A, et al (2009) Cancer-associated regulation of alternative splicing. Nat Struct Mol Biol 16: 670–676. 19448617 10.1038/nsmb.1608

[12] Parsa R, Yang A, McKeon F, Green H (1999) Association of p63 with proliferative potential in normal and neoplastic human keratinocytes. J Invest Dermatol 113: 1099–1105. 10594758 10.1046/j.1523-1747.1999.00780.x

[13] Yang A, Kaghad M, Wang Y, Gillett E, Fleming MD, et al (1998) p63, a p53 homolog at 3q27–29, encodes multiple products with transactivating, death-inducing, and dominant-negative activities. Mol Cell 2: 305–316. 9774969 10.1016/s1097-2765(00)80275-0

[14] Rocco JW, Leong CO, Kuperwasser N, DeYoung MP, Ellisen LW (2006) p63 mediates survival in squamous cell carcinoma by suppression of p73-dependent apoptosis. Cancer Cell 9: 45–56. 16413471 10.1016/j.ccr.2005.12.013

[15] Affymetrix GeneChip Exon Array Design, Technical Note.

[16] Affymetrix Identifying and Validating Alternative Splicing Events, Technical Note.

[17] Gardina PJ, Clark TA, Shimada B, Staples MK, Yang Q, et al (2006) Alternative splicing and differential gene expression in colon cancer detected by a whole genome exon array. BMC Genomics 7: 325. 17192196 10.1186/1471-2164-7-325 PMC1769375

[18] Xi L, Feber A, Gupta V, Wu M, Bergemann AD, et al (2008) Whole genome exon arrays identify differential expression of alternatively spliced, cancer-related genes in lung cancer. Nucleic Acids Res 36: 6535–6547. 18927117 10.1093/nar/gkn697 PMC2582617

[19] Wang Z, Gerstein M, Snyder M (2009) RNA-Seq: a revolutionary tool for transcriptomics. Nat Rev Genet 10: 57–63. 19015660 10.1038/nrg2484 PMC2949280

[20] Srinivasan K, Shiue L, Hayes JD (2005) Centers R, Fitzwater S, et al (2005) Detection and measurement of alternative splicing using splicing-sensitive microarrays. Methods 37: 345–359. 16314264 10.1016/j.ymeth.2005.09.007

[21] Chomczynski P (1993) A reagent for the single-step simultaneous isolation of RNA, DNA and proteins from cell and tissue samples. Biotechniques 15: : 532–534, 536–537. 7692896

[22] Chomczynski P, Sacchi N (1987) Single-step method of RNA isolation by acid guanidinium thiocyanate-phenol-chloroform extraction. Anal Biochem 162: 156–159. 2440339 10.1006/abio.1987.9999

[23] Carvalho BS, Irizarry RA (2010) A framework for oligonucleotide microarray preprocessing. Bioinformatics 26: 2363–2367. 20688976 10.1093/bioinformatics/btq431 PMC2944196

[24] Biosciences Q (2007–2009) qScript cDNA SuperMix Manual and Protocol.

[25] Sniezek JC, Matheny KE, Westfall MD, Pietenpol JA (2004) Dominant negative p63 isoform expression in head and neck squamous cell carcinoma. Laryngoscope 114: 2063–2072. 15564824 10.1097/01.mlg.0000149437.35855.4b

[26] Wang ET, Sandberg R, Luo S, Khrebtukova I, Zhang L, et al (2008) Alternative isoform regulation in human tissue transcriptomes. Nature 456: 470–476. 18978772 10.1038/nature07509 PMC2593745

[27] Klinck R, Bramard A, Inkel L, Dufresne-Martin G, Gervais-Bird J, et al (2008) Multiple alternative splicing markers for ovarian cancer. Cancer Res 68: 657–663. 18245464 10.1158/0008-5472.CAN-07-2580

[28] Venables JP, Klinck R, Bramard A, Inkel L, Dufresne-Martin G, et al (2008) Identification of alternative splicing markers for breast cancer. Cancer Res 68: 9525–9531. 19010929 10.1158/0008-5472.CAN-08-1769

[29] Chen AM, Chen LM, Vaughan A, Sreeraman R, Farwell DG, et al (2011) Tobacco smoking during radiation therapy for head-and-neck cancer is associated with unfavorable outcome. Int J Radiat Oncol Biol Phys 79: 414–419. 20399030 10.1016/j.ijrobp.2009.10.050

[30] Reis EM, Ojopi EP, Alberto FL, Rahal P, Tsukumo F, et al (2005) Large-scale transcriptome analyses reveal new genetic marker candidates of head, neck, and thyroid cancer. Cancer Res 65: 1693–1699. 15753364 10.1158/0008-5472.CAN-04-3506

[31] Wang K, Singh D, Zeng Z, Coleman SJ, Huang Y, et al (2010) MapSplice: accurate mapping of RNA-seq reads for splice junction discovery. Nucleic Acids Res 38: e178. 20802226 10.1093/nar/gkq622 PMC2952873

[32] Marinkovich MP (2007) Tumour microenvironment: laminin 332 in squamous-cell carcinoma. Nat Rev Cancer 7: 370–380. 17457303 10.1038/nrc2089

[33] Dajee M, Lazarov M, Zhang JY, Cai T, Green CL, et al (2003) NF-kappaB blockade and oncogenic Ras trigger invasive human epidermal neoplasia. Nature 421: 639–643. 12571598 10.1038/nature01283

[34] McGowan KA, Marinkovich MP (2000) Laminins and human disease. Microsc Res Tech 51: 262–279. 11054876 10.1002/1097-0029(20001101)51:3<262::AID-JEMT6>3.0.CO;2-V

[35] Cheng YS, Champliaud MF, Burgeson RE, Marinkovich MP, Yurchenco PD (1997) Self-assembly of laminin isoforms. J Biol Chem 272: 31525–31532. 9395489 10.1074/jbc.272.50.31525

[36] Moller-Levet CS, Betts GN, Harris AL, Homer JJ, West CM, et al (2009) Exon array analysis of head and neck cancers identifies a hypoxia related splice variant of LAMA3 associated with a poor prognosis. PLoS Comput Biol 5: e1000571. 19936049 10.1371/journal.pcbi.1000571 PMC2773424

[37] Okumura M, Yamakawa H, Ohara O, Owaribe K (2002) Novel alternative splicings of BPAG1 (bullous pemphigoid antigen 1) including the domain structure closely related to MACF (microtubule actin cross-linking factor). J Biol Chem 277: 6682–6687. 11751855 10.1074/jbc.M109209200

[38] Tamai K, Sawamura D, Do HC, Tamai Y, Li K, et al (1993) The human 230-kD bullous pemphigoid antigen gene (BPAG1). Exon-intron organization and identification of regulatory tissue specific elements in the promoter region. J Clin Invest 92: 814–822. 8349819 10.1172/JCI116655 PMC294919

[39] Herold-Mende C, Kartenbeck J, Tomakidi P, Bosch FX (2001) Metastatic growth of squamous cell carcinomas is correlated with upregulation and redistribution of hemidesmosomal components. Cell Tissue Res 306: 399–408. 11735040 10.1007/s004410100462

[40] Hamill KJ, Hopkinson SB, DeBiase P, Jones JC (2009) BPAG1e maintains keratinocyte polarity through beta4 integrin-mediated modulation of Rac1 and cofilin activities. Mol Biol Cell 20: 2954–2962. 19403692 10.1091/mbc.E09-01-0051 PMC2695802

