# Supplementary figures and images for: Expression Microarray Analysis Reveals Alternative Splicing of LAMA3 and DST Genes in Head and Neck Squamous Cell Carcinoma
Source: PLoS One. 2014 Mar 27;9(3):e91263. doi: 10.1371/journal.pone.0091263 (PMC3967989; doi:10.1371/journal.pone.0091263)

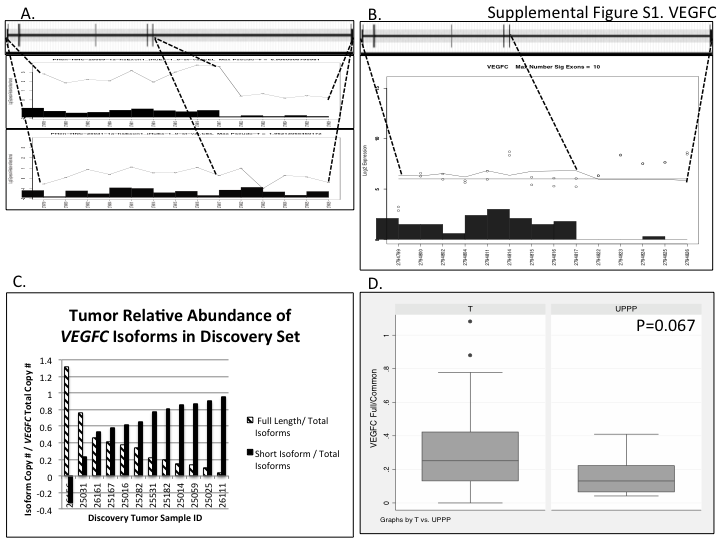

Supplement: Figure S1 — VEGFC . (TIFF) [file pone.0091263.s001.tif]

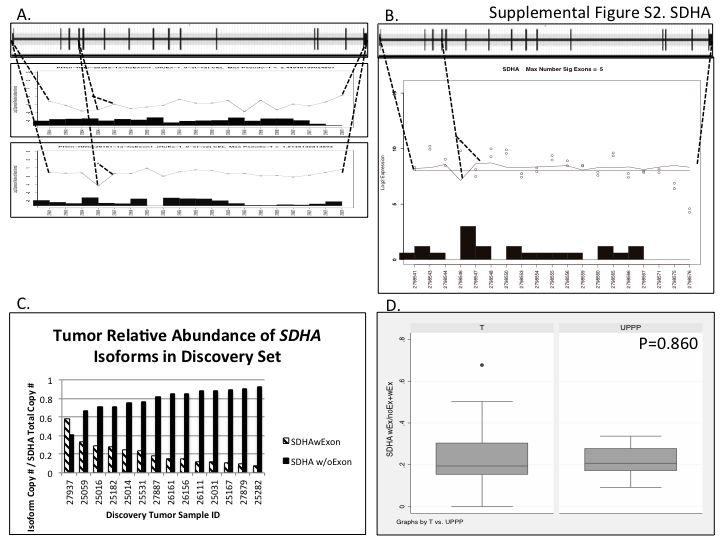

Supplement: Figure S2 — SDHA . (TIFF) [file pone.0091263.s002.tif]

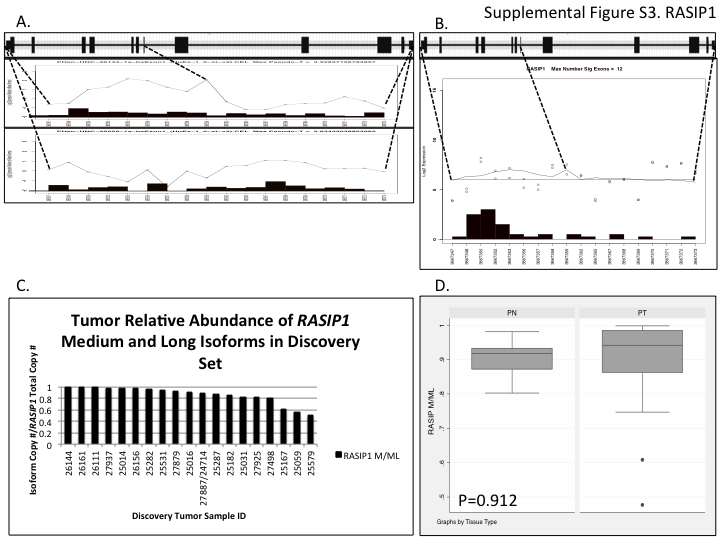

Supplement: Figure S3 — RASIP1 . (TIFF) [file pone.0091263.s003.tif]

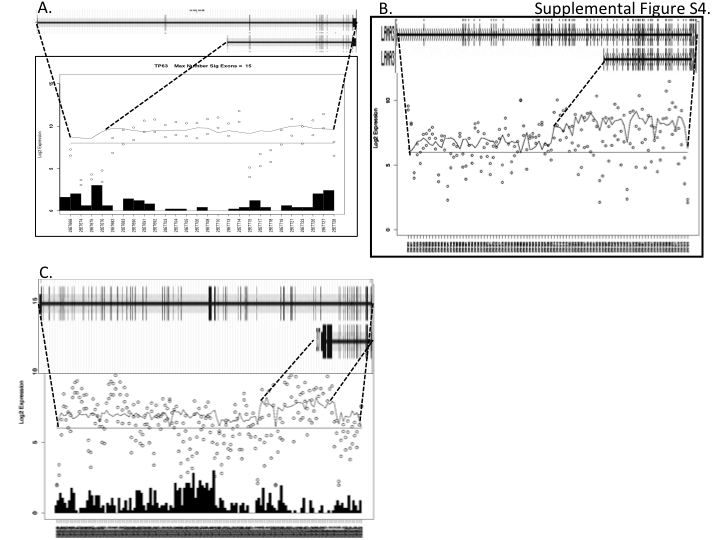

Supplement: Figure S4 — Visual screening begins with a graphical comparison of the discovery tumor cohort and UPPP sample expression array data. A,TP63 gene. Comparing the mean expresssion of each probeset in the entire discovery tumor set to the mean in the entire discovery UPPP set. The mean probeset signals for the UPPP sample set have been zeroed (horizontal line), with adjustment of tumor cohort mean probeset signals (oscillating line), log2 scale. B, LAMA3 gene. Comparing the mean expresssion of each probeset in the entire discovery tumor set to the mean in the entire discovery UPPP set. The mean probeset signals for the UPPP sample set have been zeroed (horizontal line), with adjustment of tumor cohort mean probeset signals (oscillating line), log2 scale. C, DST gene. Comparing the mean expresssion of each probeset in the entire discovery tumor set to the mean in the entire discovery UPPP set. The mean probeset signals for the UPPP sample set have been zeroed (horizontal line), with adjustment of tumor cohort mean probeset signals (oscillating line), log2 scale. (TIFF) [file pone.0091263.s004.tif]
